# Fully Automated Trimethylsilyl (TMS) Derivatisation Protocol for Metabolite Profiling by GC-MS

**DOI:** 10.3390/metabo7010001

**Published:** 2016-12-29

**Authors:** Erica Zarate, Veronica Boyle, Udo Rupprecht, Saras Green, Silas G. Villas-Boas, Philip Baker, Farhana R. Pinu

**Affiliations:** 1 School of Biological Sciences, University of Auckland, Private Bag 92019, Auckland 1010, New Zealand; e.zarate@auckland.ac.nz (E.Z.); saras.green@auckland.ac.nz (S.G.); s.villas-boas@auckland.ac.nz (S.G.V.-B.); 2 The Liggins Institute, University of Auckland, Private Bag 92019, Auckland 1010, New Zealand; v.boyle@auckland.ac.nz (V.B.); philip.baker@le.ac.uk (P.B.); 3 Lasersan Australasia Pty Ltd., Robina QLD 4226, Australia; udo@lasersan.com; 4 College of Medicine, Biological Sciences and Psychology, University of Leicester, Leicester LE1 7RH, UK; 5 Sustainable Production, The New Zealand Institute for Plant & Food Research Limited, Private Bag 92169, Auckland 1142, New Zealand

**Keywords:** metabolomics, matrix, automation, sample preparation, sugars, amino acids, organic acids

## Abstract

Gas Chromatography-Mass Spectrometry (GC-MS) has long been used for metabolite profiling of a wide range of biological samples. Many derivatisation protocols are already available and among these, trimethylsilyl (TMS) derivatisation is one of the most widely used in metabolomics. However, most TMS methods rely on off-line derivatisation prior to GC-MS analysis. In the case of manual off-line TMS derivatisation, the derivative created is unstable, so reduction in recoveries occurs over time. Thus, derivatisation is carried out in small batches. Here, we present a fully automated TMS derivatisation protocol using robotic autosamplers and we also evaluate a commercial software, Maestro available from Gerstel GmbH. Because of automation, there was no waiting time of derivatised samples on the autosamplers, thus reducing degradation of unstable metabolites. Moreover, this method allowed us to overlap samples and improved throughputs. We compared data obtained from both manual and automated TMS methods performed on three different matrices, including standard mix, wine, and plasma samples. The automated TMS method showed better reproducibility and higher peak intensity for most of the identified metabolites than the manual derivatisation method. We also validated the automated method using 114 quality control plasma samples. Additionally, we showed that this online method was highly reproducible for most of the metabolites detected and identified (RSD < 20) and specifically achieved excellent results for sugars, sugar alcohols, and some organic acids. To the very best of our knowledge, this is the first time that the automated TMS method has been applied to analyse a large number of complex plasma samples. Furthermore, we found that this method was highly applicable for routine metabolite profiling (both targeted and untargeted) in any metabolomics laboratory.

## 1. Introduction

Because of the recent advancements in analytical instrumentations and data analysis platforms, metabolome analysis has been improved tremendously in the last 10 years. It is now possible to detect and identify thousands of metabolites in a single run [1,2,3]. Although, initially, metabolomics had been applied mostly as a data-driven hypothesis generating approach in an un-targeted manner, recently both targeted and un-targeted metabolomics are becoming more popular to resolve many research questions [3,4,5,6]. Among different techniques of metabolite analysis, metabolite profiling is the oldest and most widely used in life sciences, and focuses on the study of a large group of metabolites related to different metabolic pathways [7]. Gas Chromatography-Mass Spectrometry (GC-MS) was the first instrumentation used for metabolite profiling of human blood and urine by Horning in 1971 [8]. Since then, GC-MS has been applied to determine the metabolite profiles of many biological samples including plants, animals, and microorganisms [9,10,11,12,13,14]. As GC-MS has been used for metabolite profiling for over five decades, many protocols for machine set-up and maintenance are already available [9]. The pipelines for sample preparation, analysis, chromatogram evaluation, and interpretation by GC-MS are also well established allowing identification (using in-house and commercial MS libraries) and quantification of over hundreds of metabolites in any biological sample [15,16]. One major advantages of GC-MS is that it has a relatively comprehensive range of coverage, thus permitting the analysis of organic and amino acids, sugars (usually < 3 mono-saccharides in length), sugar alcohols, fatty acids, and phosphorylated compounds in various samples [9,17]. However, an additional step of sample preparation, derivatisation is required to convert the polar/semi-polar metabolites into their volatile counterparts, to facilitate analysis by GC-MS.

Many derivatisation methods are available, and alkylation and silylation are the most widely used protocols in metabolome analysis and metabolite profiling [16,18]. Although the alkylation (using chloroformates, especially methyl chloroformate as the derivatising agents) offers many advantages, this method cannot derivatise sugars and sugar alcohols [17,18]. In contrast, silylation offers coverage of a broad range of metabolites. Silylation is the classical derivatisation method that introduces a silyl group [–Si(CH_3_)_3_–] to the metabolite by replacing the active hydrogen (e.g., –OH, –SH, –NH_4_^+^, –COOH) to generate stable, more volatile, and less polar derivatives of the parental metabolites [14,18]. Silylation is comparatively safe, easy to use and generally one or two reaction steps are required [19]. The most common silylation reagents are *N*,*O*-bis(trimethylsilyl) trifluroacetamide (BSTFA) and *N*-Methyl-*N*-(trimethylsilyl) trifluoroacetamide (MSTFA) [19]. The reagents for silylation are very sensitive to moisture, so anhydrous conditions are required and some samples with high sugar concentrations pose a challenge because of the difficulty in achieving complete dryness. Additionally, the reaction mixture is not separated from the derivatives and the sample injected into the GC inlet and then into the column consists of derivatives and residual reagents, which may cause damage to the GC column, reducing its life time [18]. However, in order to increase the column longevity, back-flush setting can be used to limit the amount of derivatising reagents from entering into the column. Moreover, this derivatisation process takes longer than 1 h, and the heating process to ensure the optimum reaction condition can be destructive to some thermo-labile compounds. However, a microwave-assisted method of silylation is available, which reduces the reaction time from 1 h to 3–5 min [14,19,20]. However, microwaves can cause the degradation of many unstable metabolites in biological samples. 

Most of the published trimethylsilyl (TMS) protocols make use of off-line derivatisation [18,21]. It is well known that the majority of TMS derivatives are unstable; derivatisation of more than a small batch of samples at once will result in variability among the samples injected at different times [18]. To overcome this, an automated online method can be beneficial as it allows immediate derivatisation of samples just prior to the injection [22]. The integrity and reproducibility of the data can also be increased using an automatic online derivatisation method [12]. Robotic autosamplers are available that can perform derivatisation on-line, followed immediately by GC-MS injection. In conjunction with the commercial software Maestro, available from Gerstel GmbH (Muehlheim, Germany), automatically overlapping derivatisation and GC-MS acquisition can be performed. Our main aim in this study was to develop a fully automated method for the metabolite profiling of a wide range of biological samples using the Gerstel Maestro software. Here we compare the data obtained from both manual (off-line) and automated (on-line) TMS derivatisation methods. We also validated the automatic method by using over 114 plasma samples run over nine weeks.

## 2. Results and Discussion

### 2.1. Comparison between Automated and Manual TMS Derivsation

**Figure 1:**
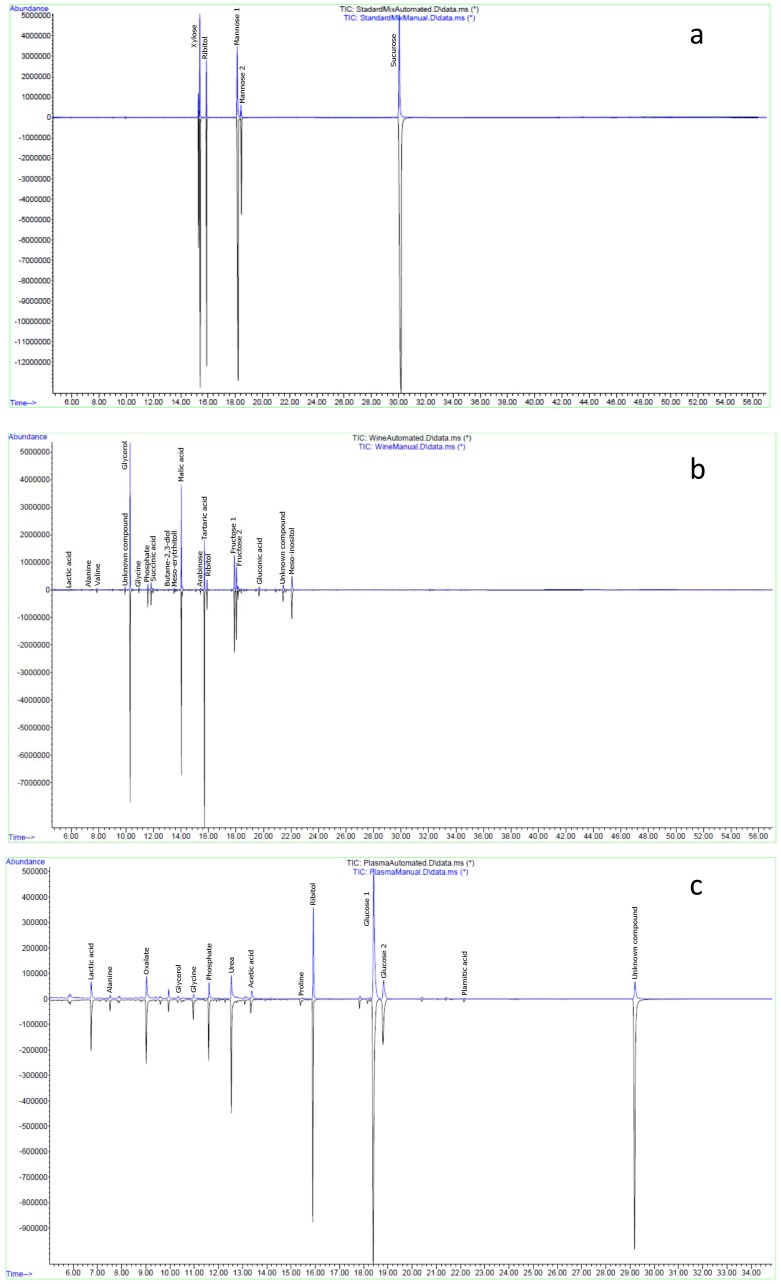
Chromatograms showing the difference between automated and manual TMS derivatisation protocols for standard mix (**a**); wine (**b**); and plasma (**c**) samples.

In this study, automated derivatisation undertaken by preparing a batch of samples using a similar method as in the manual protocol. However, a few modifications were adopted to acclimate the method to the MPS2 MultiPurpose Sampler (Gerstel, Muehlheim, Germany). For the manual derivatisation, two different incubation temperatures (30 °C and 37 °C) are usually used [18,21]. For the automated method, it was not possible to change the temperatures in the agitator within a few minutes. For this reason, only one temperature (37 °C) was used to allow the completion of the derivatisation process [12], which was evident from the data shown in Figure 1, and Table 1 and Table 2. One of the most positive aspects of this automated method was that we optimised it by using a smaller amount of reagents (40 µL instead of 80 µL required for manual derivatisation). This indeed allowed us to save 50% of reagent costs. Once the sample was derivatised, the injection took place two hours later. This step was optimised so that plasma samples could be completely derivatised and was easily achieved using the Gerstel Maestro software that allowed sample derivitisation overlapping with the GC-MS run time. 

Despite these small changes, as compared to the manual TMS derivatisation, we found that most of the metabolites showed better reproducibility when automated derivatisation was performed in three different matrices (Figure 1 and Table 2). Interestingly, the total number of metabolite detections and total ion chromatogram (TIC) areas of both wine and plasma samples were significantly higher when the samples were derivatised using automated method (Table 1). However, Abbiss et al. [12] observed slightly different results between rat urine samples derivatised automatically but injected immediately after derivatisation (in-time) and samples derivatised in batch (batch). They found that both the number of metabolites detected and overall intensity (TIC) and of in-time samples were lower compared to the batch samples. On the contrary, our results clearly indicate that the automated method was more effective for our samples than the manual one.

Comparison of the chormatograms of the samples derivatised with these two different methods is also presented in Figure 1 and the same metabolites were identified in both methods. Moreover, it was clearly visible from the overlaid chromatograms that the peak heights for most of the metabolites were considerably higher (>50%) in samples that were derivatised using the online automated method rather than the offline manual method (Figure 1). This observation again suggests the superiority of the automated derivatisation protocol over the manual version.

It is noteworthy that this automated method allowed us to inject the samples precisely at the same stage of derivatisation without any further delay or waiting on the autosampler. On the contrary, while derivatising manually, there was a variation in waiting time between injecting the first and latter samples, which affects the reproducibility and quality of the final data [12,22]. Many metabolites are degraded or new derivatives (most probably breakdown products) may develop while the sample is waiting on the autosampler to be injected. Therefore, an automated online method is preferable as it allows a better control of the whole derivatisation and injection of samples. It increases the overall throughput because of the overlapping of samples. Thus, this method saves researcher time and also reduces the variability between samples injected at different time intervals [12,22].

The chromatograms presented in Figure 1 show that two peaks were found from the TMS derivatisation of sugars (e.g., glucose and mannose), which was in accordance to previously published literature on this method [9,13,14,18]. TMS derivatisation is carried out in two steps. Methoximation of carbonyl groups of sugar, sugar derivatives and other metabolites to prevent the ring formation and also to reduce the number of sterioisomers [14]. This step is followed by the replacement of active hydrogens by TMS reagent [14]. However, multiple peaks are usually produced when the silylation is incomplete. Other factors, such as temperature, the time of derivatisation, and the amount of derivatising reagents also may contribute to the formation of multiple peaks. This is a common problem, well known within the metabolomics community that can be dealt easily. It is usually recommended to sum up the peak areas of the multiple peaks if the response factors are not the same [18]. In our case, we found a very good correlation (R^2^ > 0.80) between the two peaks of the sugars (glucose, fructose and galactose, data not shown) and we reported the abundance of both of the peaks (Table 2 and Table 3). All these metabolites (including multiple peaks) were positively identified using our in-house MS library that contains information about the retention time, reference ion, and mass fragments obtained from metabolite standards. Moreover, all the samples from both manual and automated derivatisation were run in “scan” mode, thus both targeted and untargeted metabolite profiling data can be obtained from them. However, here we showed the results of different important groups of primary metabolites to compare between two different derivatisation protocols (manual and automated).

In metabolomics, data reproducibility is usually reported as Relative Standard Deviation (RSD, expressed as %) and the maximum acceptance tolerance of 30% is reported for GC-MS metabolomics studies [23]. RSD of less than 10–15% is considered good reproducibility [13,16]. When we compared the data obtained from three different matrices (standard mixes, wines, and plasmas) using manual and automated TMS derivatisation, we found good reproducibility (RSD < 20%) for most of the compounds identified, with a few exceptions. The internal standard used (ribitol) showed similar and very high reproducibility (RSD ≤ 2–3%) in both automated and manual derivatisation for the standard mix of sugars, suggesting that this matrix does not have a big effect on the internal standard chosen. In contrast, the automated method showed a much better reproducibility for ribitol than manual method when wine and plasma samples were analyzed (Table 1). These results indicate that the automated method was more reproducible for the internal standard compared to the manual method for different matrixes. 

Very high reproducibility (RSD < 10%) was found for the seven sugars present in the standard mixes. This reproducibility was slightly better for two of those sugars (mannose and arabinose) when the automated method was used than with the manual one (Table 1). The reproducibility of metabolites in the wine samples, however, showed different results when the two methods were compared. The amino acids identified indicated slightly better reproducibility when the manual method was used. The exception was glycine, which showed a better reproducibility when the automated method was used (RSD = 10%) compared with the manual one (RDS = 19%). In contrast, most of the sugars had improved reproducibility when the automated method was used (RSD < 10%). Unfortunately, the reproducibility decreased for most of the organic acids identified in wines when the automated method was used, suggesting that the manual derivatisation might be a better choice if these are the metabolites of interest in any study. However, we generally use a methylchloroformate (MCF) based derivatisation protocol for the simultaneous determination of amino and organic acids [10,18]. We also prefer to use TMS method for the analysis of sugars and sugar alcohols [18,21] and the automated TMS method showed good reproducibility for these types of metabolites in wine samples. Plasma samples are known to be one of the most difficult matrices to work with, because of the presence of high amount of proteins and large molecular weight lipids. After drying, these samples become very adhesive to the bottom of the insert, which causes a significant challenge in dissolving the samples with the first reagent. For this reason, a two-hour delay was added to the method to allow the samples to be dissolved properly and this improved the overall derivatisation process. Even though the internal standard showed a better reproducibility when the automated method was used, the amino acids did not show the same results (Table 1). The only amino acid that showed better reproducibility using the automated method was serine, decreasing the RSD from 22% to 9%. Sugars showed similar reproducibility when these two methods were compared (RSD < 12%). However, allose- and meso-inositol showed much better reproducibility when the automated method was performed (RSD 22% and 21% reduced to 7% and 12%, respectively). The reproducibility for organic acids indicated ambiguous results. While lactic acid maintained rather high reproducibility (RSD ~ 5%), the variability for oxalic acid increased abruptly from 8% in the manual method to 22% for the automated method. However, it is noteworthy that both lactic and oxalic acids are poor indicators of reproducibility as they are the early eluting compounds and highly affected by the changes in temperature. We used only one temperature (37 °C) for the automated method, while the traditional TMS derivatisation makes use of two different temperatures (e.g., 30 °C for methoximation and 37 °C for silylation). This could explain the variability between the results we observed from the methods for both of these early eluting organic acids.

In summary, the automated method provided either similar or better reproducibility for most of the metabolites. We also observed an improved reproducibility when we used the automated method for sugars and sugar alcohols, which are our metabolites of interest from the TMS method. 

### 2.2. Validation of Automated TMS Method Using Plasma QC Samples over Nine Weeks

Once the automated TMS method was optimised successfully, we analysed over 114 quality control (QC) plasma samples over nine weeks to check the overall changes in metabolite profiles over this period of time. Plasma samples are known to contain over thousands of metabolites and many of them are covalently bound to proteins [24]. Therefore, liquid extraction of plasma samples was performed to overcome the matrix effects by dissociating metabolites and removing proteins, phospholipids, and large molecular weight lipids. QC samples were prepared by pooling a small aliquot of all samples (see Methods and Materials Section); thus, it was expected that the data obtained from these samples would be more or less similar. However, we cannot exclude the possibility of a small amount of variation due to the changes in analytical parameters of the instrument over the time including ion source cleaning, column cut, liner changing, and system venting. These QC samples were analysed periodically, along with a large batch of plasma samples, for the quality assurance and signal correction purposes [23]. No column cutting and ion source cleaning was performed while analysing the samples. We detected over 180 features in the plasma samples and positively identified over 35 metabolites using our in-house MS library that contains information about reference ion and retention time. However, here we report only the major metabolites [21] belonging to different groups present in the plasma samples (Table 3). Although the RSD values of most of these metabolites were below 30%, a specific group of metabolites, fatty acids and their derivatives, did not show good reproducibility (RSD > 40%); this indicates that TMS might not be a suitable method for the analysis of these metabolites. Moreover, the intensity of these metabolites was quite low, which may result in difficulties in integrating these peaks properly (due to the deconvolution) [12]. Although amino acids showed a mixed trend of reproducibility when we compared the data of different matrices derivatised by the manual and automated TMS methods, all the eight identified amino acids in plasma QC samples showed reproducibility within an acceptable range (RSD < 30%). Most importantly, a very good reproducibility (RSD < 20%). was obtained for most of the sugars, sugar alcohols, organic acids, phosphate, and urea, which are our metabolites of interest using the TMS method (Table 2).

**Figure 2:**
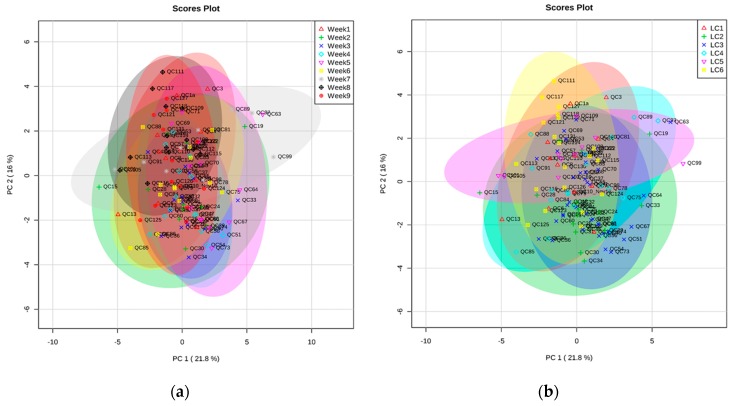
Two dimensional projections of Principal Component Analysis (PCA) using 23 major metabolites identified using automatic trimetylsilil (TMS) derivatisation of plasma samples. Here, LC denotes the GC inlet liner change. (**a**) week to week variation; (**b**) GC inlet liner change.

We also performed a Principal Components Analysis (PCA) using these 23 major metabolites (Figure 2) to verify the changes in metabolite profiles of QC samples from week to week and also to determine if GC inlet liner change had any effect on the quality of the data or not. Every week, we analysed at least 10–15 QC plasma samples depending on the day-to-day sequence. We did not see any clustering pattern on a weekly basis; rather, most of the samples grouped together, indicating their similarity in metabolite profiles. (Figure 2b). However, there were a few outliers. It is important to note that there could be other technical parameters (e.g., extraction of samples, pipetting) responsible for the minor changes that occurred between the samples. Overall, our data clearly indicated that metabolite profiles of 114 QC plasma samples were very reproducible over the nine weeks, suggesting a successful optimisation of the automated TMS method. 

In addition to determining the weekly difference, we choose another parameter, the change in GC inlet liner, in the PCA. We know from our experience that liner change can affect the sensitivity (e.g., reduction in peak intensity) of the GC instrument significantly if samples are running constantly without any maintenance. We previously observed that, if the liner is not changed in every 100–200 samples (depending on the cleanliness of the samples), then reproducibility between samples reduces considerably (data not shown). During the time these 114 QC plasma samples were run (along with a large batch of experimental plasma samples), we changed the liner six times over nine weeks. Figure 2a clearly shows that metabolite profiles of the QC samples did not vary depending on the liner change. Therefore, we were confident that our automated TMS method generated a good set of QC data that could be used for the signal correction of a large batch of experimental plasma samples. 

## 3. Materials and Methods

### 3.1. Chemicals

All the chemicals used for this study were of analytical grade. Internal standard and reagents for GC-MS, D-ribitol, *N*-methyl-*N* (trimethylsilyl) trifluoroamide (MSTFA), pyridine, and all other metabolite standards, were purchased from Sigma-Aldrich (St. Louis, MO, USA). Methoxyamine hydrochloride was obtained from Fluka (Steinheim, Switzerland), anhydrous sodium sulphate from BDH chemicals (Poole, UK), and acetone from Biolab (Scoresby, Australia). Methanol, sodium hydroxide and sodium bicarbonate were purchased from Merck (Darmstadt, Germany). All the chemical solutions were prepared using Grade 1 water (Barnstead ^®^ NANOpure Diamond™ Water Purification System, Waltham, MA, USA) or absolute ethanol (Univar, Ajax Finechem, Auckland, New Zealand).

### 3.2. Sample Preparation

We used three different types of matrices to compare the results between manual and automated derivatisation including standard metabolite mixes (10 mM of ribitol, alanine, leucine, lysine, tryptophan, arabinose, glucose, fructose, mannose, galactose, xylose, sucrose, glycerol, ferrulic acid, 2-hydroxybutyric acid, and urea), wines, and plasma.

#### 3.2.1. Preparation of Standard Mixes and Wine Samples

Frozen wine samples (20 µL) and standard mix (20 µL) were thawed, vortexed, and transferred to an insert in a GC-vial, separately. The internal standard, D-ribitol, (20 µL, 10 mM) and methanol (60 µL) were added to the inserts. The samples were vortexed a second time. After that, they were dried for 4 h using a Speed-Vac Concentrator with a Refrigerated Vapor Trap (SC250EXPP2-115, Thermo Scientific, New Zealand). The samples were then transferred to an evacuated desiccator with phosphorous pentoxide overnight to dry them completely.

#### 3.2.2. Extraction of Metabolites from Plasma Samples and Preparation of Quality Control (QC) Samples

An amount of 20 µL of internal standard, ribitol, was added to 150 µL of plasma. Plasma samples were collected from participants of the MAVIDOS trial at 34 weeks’ gestation [25]. Plasma samples were dried in a Speed-Vac Concentrator with a Refrigerated Vapor Trap (SC250EXPP2-115, Thermo Scientific, New Zealand). Liquid extraction was performed by adding 500 µL of cold 50:50 methanol:water. After mixing properly, the vial was centrifuged for 5 min at 3500 rpm at −4 °C.

The process was repeated with 500 μL cold 80:20 methanol:water followed by centrifugation. Samples were again dried in the Speed-Vac Concentrator. QC samples were prepared by pooling aliquots (500 µL) from all extracted serum samples. QC samples were dried again completely using the Speed-Vac Concentrator and then kept at an evacuated desiccator prior to the analysis.

### 3.3. Trimethylsilyl (TMS) Derivatisation Protocols

#### 3.3.1. Automated TMS Derivatisation

**Figure 3:**
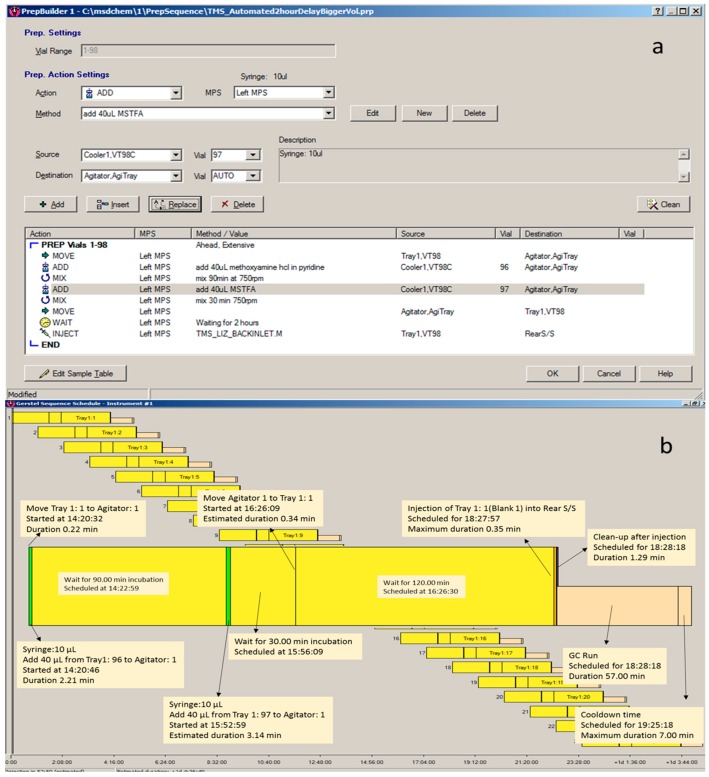
Screen shots from the Gerstel Maestro software showing the method for the sample preparation and injection step by step (**a**) and the overlapping the sequence (**b**).

An automatic TMS derivatisation protocol was used to analyses the sugars, sugar alcohols, and other derivatives in plasma samples, using a GERSTEL MAESTRO 1.4 software (Muehlheim, Germany). A sequence was created to run the samples automatically (Figure 3).

The reagents, methoxyamine hydrochloride in pyridine (2 g/100 mL) and MSTFA, were placed into 2 mL amber GC vials and kept in the cool tray (4 °C). The vials with dried extracted plasma samples were capped with metal caps and placed on the GC tray 1. To start the derivatisation process, a vial with the sample was moved to the agitator and 40 µL of methoxyamine hydrochloride in pyridine was added to dissolve the dried sample. The sample was mixed for 90 min at 750 rpm and 37 °C in the agitator. 40 µL of the second reagent, MSTFA (*N*-methyl-*N*-(trimethylsilyl trifluoroacetamide)) was then added to the sample and mixed for 30 min again at 750 rpm and 37 °C. Two hours after finishing the derivatisation, the vial was moved back to tray 1 and remained there for 2 h before the injection to finish the reaction.

An Agilent GC 7890A coupled to a MS 5975C (Agilent Technologies, Santa Clara, CA, USA) with a quadrupole mass selective detector (electron ionization) with a split/splitless inlet (according to the protocol described by Villas-Boas et al., 2011) was used to analyse the metabolite profile. One microliter (1 µL) of the sample was injected using the Gerstel MPS2 MultiPurpose Sampler into a glass split/splitless 4 mm ID straight inlet liner packed with deactivated glass wool. The inlet was set to 230 °C, with a split ratio of 25:1, pressure 99.26 kPa, a column flow of 1.3 mL/min constant flow mode, giving a calculated average initial linear velocity of 39 cm/s. The column (5 m guard column) was a fused silica ZB-1701 30 m long, 0.25 mm i.d., 0.15 µm stationary phase (86% dimethylpolysiloxane, 14% cyanopropylphenyl, Phenomenex, Torrance, CA, USA). The carrier gas was ultra-high purity grade helium (99.9999%, BOC). GC oven temperature programming started isothermally at 70 °C for 5 min, was increased 10 °C/min to 179 °C; then increased 0.5 °C/min to 180 °C, held 2 min, then increased 10 °C/min to 220 °C, held 1 min, then increased 2.5 °C/min to 265 °C, held 1 min, then increased 10 °C/min to 280 °C, held 1 min, and finally increased 1 °C/min to 290 °C, before being held for 0.6 min. The transfer line to the mass spectrometer detector (MSD) was maintained at 250 °C, the source at 230 °C and quadrupole at 150 °C. The detector was turned on 4.5 min into the run. The detector was run in positive-ion, electron-impact ionisation mode, at 70 eV electron energy, with electron multiplier set with no additional voltage relative to the autotune value. Mass spectra were acquired in scan mode from 40 to 650 amu, with a detection threshold of 100 ion counts.

#### 3.3.2. Manual TMS derivatisation

The manual TMS derivatisation was performed following the protocol published in Pinu et al. [21] and the derivatised samples were injected into an Agilent GC 7890 coupled to a MSD 5975 (Agilent Technologies, Santa Clara, CA, USA) with a quadrupole mass selective detector (Electron Ionisation) operated at 70 eV. The column used for the analysis of TMS-derivatized samples was a Zebron ZB-1701 (Phenomenex, Torrance, CA, USA), 30 m × 250 µm (internal diameter) × 0.15 µm (film thickness), with a 5-m guard column. The MS was operated in scan mode, where scanning started after 5 min (mass range 40 to 650 atomic mass unit (a.m.u ) at 1.47 scans/s). GC-MS parameters are already described in Villas-Boas et al [18].

### 3.4. Data Mining and Analysis

GC-MS data mining was carried out according to the methods of Aggio et al. [26] and Pinu et al. [21] using in-house R-based software and scripts (Version 3.0.1). ANOVA was performed using in-house R scripts. Principal Component Analysis (PCA) was performed using a web interface, Metaboanalyst 3.0 (http://www.metaboanalyst.ca), created by the University of Alberta, AB, Canada [27]. For other data analysis, Microsoft^®^ Excel 2007 was also used.

## 4. Conclusions 

Automated sample preparation and derivatisation protocols are gaining enormous popularity [12,22,28] as these steps provide a simplified workflow by reducing analysis time significantly and also by increasing the integrity and reproducibility of the metabolite profiling data. We optimised an automated trimethylsilyl (TMS) derivatisation protocol using MSFTA as derivatising agent. With the help of Maestro, software available from Gerstel GmbH, this method allowed us to overlap and inject samples at the same stage of derivatisation. Therefore, this method could be used for the analysis of wide range of biological samples with different matrices with improved throughputs. Our results clearly indicated that most of the metabolites showed either similar or better reproducibility when we compared manual and automated TMS derivatisation methods. Moreover, the metabolite profiles of QC plasma samples did not vary significantly over a nine week run time or even when GC inlet liners were changed, suggesting successful optimisation of the automated method. This is the first time automated TMS method was used for the analysis of large number of plasma samples. We highlight the advantage of the automated derivatisation over the manual method, resulting in a complete automation and very robust analysis, time savings for the researcher, and also cost reductions. 

## Figures and Tables

**Table 1:** Number of detections and total ion chromatogram (TIC) areas obtained from both automated and manual trimethylsilyl (TMS) derivatisation methods in wine and plasma samples.

	Wine (*n* = 8)		Plasma (*n* = 4)	
	Automated	Manual	Automated	Manual
**Average No. of Detection**	240 ± 25 ^b^	157 ± 18	167 ± 17 ^a^	114 ± 17
**Average TIC**	112,058 ± 15,245 ^b^	40,907 ± 3125	12,717 ± 3015	10,424 ± 2255

Here, ^a^ indicates *p*-value < 0.05 and ^b^ indicates *p*-value < 0.001.

**Table 2:** Comparison between automatic (on-line) and manual (off-line) trimethylsilyl (TMS) derivatisation in different matrices.

		Standard Mix (RSD%, *n* = 5)		Wine (RSD%, *n* = 8)		Plasma (RSD%, *n* = 3)	
	Metabolites	Automated	Manual	Automated	Manual	Automated	Manual
**Internal Standard**	Ribitol	2	3	7	15	3	5
**Amino Acids**	Alanine	9	19	13	11	10	3
	Glycine	NI	NI	10	19	19	7
	Leucine	7	21	23	20	45	18
	Isoleucine	NI	NI	ND	ND	54	12
	Lysine	11	ND	ND	ND	ND	ND
	Proline	NI	NI	13	10	23	11
	Serine	NI	NI	ND	ND	9	22
	Threonine	NI	NI	ND	ND	13	13
	Tryptophan	14	ND	ND	ND	ND	ND
	Valine	NI	NI	16	15	18	28
**Sugars and Sugar Alcohols**	Allose	NI	NI	8	12	7	22
	Arabinose	2	5	9	13	ND	ND
	Cellobiose	NI	NI	5	13	ND	ND
	Fructose 1	3	5	7	8	ND	ND
	Fructose 2	3	4	7	9	ND	ND
	Galactose	3	3	8	9	7	4
	Glycerol	5	8	7	13	4	4
	Glucose 1	3	2	ND	ND	4	2
	Glucose 2	5	3	ND	ND	5	4
	Mannose 1	3	6	8	9	ND	ND
	Mannose 2	6	5	8	12	ND	ND
	Meso-erythritol	NI	NI	6	17	ND	ND
	Meso-inositol	NI	NI	9	6	12	21
	Sorbose	NI	NI	7	8	ND	ND
	Sucrose	6	6	ND	ND	ND	ND
	Xylose	5	5	2	3	ND	ND
**Organic Acids**	2-hydroxybutyric acid	5	11	ND	ND	ND	ND
	Glucaric acid	NI	NI	9	6	ND	ND
	Gluconic acid	NI	NI	27	8	ND	ND
**Others**	Ferulic acid	5	9	ND	ND	ND	ND
	Lactic acid	NI	NI	23	17	5	4
	Malic acid	NI	NI	8	9	ND	ND
	Oxalic acid	NI	NI	38	27	22	8
	Butane-2,3-diol	NI	NI	7	14	ND	ND
	Phosphate	NI	NI	11	10	6	7
	Urea	8	13	ND	ND	9	11

NI = not included, ND = not detected, RSD = Residual Standard Deviation.

**Table 3:** Major metabolites present in plasma samples and their Residual Standard Deviation (RSD) over nine weeks.

Metabolite	Average Relative Abundance in Plasma Samples (*n* = 114)	Average RSD over Nine Weeks (%; *n* = 114)
**Sugars and sugar alcohols**		
Galactose	0.011 ± 0.002	14
Glucose peak 1	1.368 ± 0.134	10
Glucose peak 2	0.204 ± 0.030	15
Glycerol	0.038 ± 0.011	28
Meso-inositol	0.010 ± 0.001	12
**Amino acids**		
Alanine	0.108 ± 0.027	25
Glycine	0.076 ± 0.018	23
Lysine	0.011 ± 0.003	29
Proline	0.079 ± 0.022	27
Serine	0.006 ± 0.001	20
Threonine	0.012 ± 0.002	20
Valine	0.062 ± 0.016	26
**Organic acids**		
2-hydroxybutyic acid	0.018 ± 0.004	24
Isocitric acid	0.028 ± 0.004	14
Lactic acid	0.701 ± 0.099	14
**Fatty acids**		
Linoleic acid	0.004 ± 0.0016	42
Oleic acid	0.007 ± 0.0031	43
Palmitoleic acid	0.012 ± 0.0064	56
Stearic acid	0.001 ± 0.0005	51
**Others**		
Cholesterol	0.076 ± 0.036	47
Phosphate	0.869 ± 0.122	14
Urea	0.887 ± 0.121	14
